# Image analysis for the automatic phenotyping of *Orobanche cumana* tubercles on sunflower roots

**DOI:** 10.1186/s13007-021-00779-6

**Published:** 2021-07-21

**Authors:** A. Le Ru, G. Ibarcq, M.- C. Boniface, A. Baussart, S. Muños, M. Chabaud

**Affiliations:** 1 FRAIB, Castanet-Tolosan, France; 2 LIPME, Université de Toulouse, INRAE, CNRS, Castanet-Tolosan, France; 3 MAS Seeds, Haut Mauco, France

**Keywords:** Broomrape, *Orobanche*, Sunflower, Image analysis, Macro, Phenotyping, Raspberry Pi, Tubercle, Nodule

## Abstract

**Background:**

The parasitic plant *Orobanche cumana* is one of the most important threats to sunflower crops in Europe. Resistant sunflower varieties have been developed, but new *O. cumana* races have evolved and have overcome introgressed resistance genes, leading to the recurrent need for new resistance methods. Screening for resistance requires the phenotyping of thousands of sunflower plants to various *O. cumana* races. Most phenotyping experiments have been performed in fields at the later stage of the interaction, requiring time and space. A rapid phenotyping screening method under controlled conditions would need less space and would allow screening for resistance of many sunflower genotypes. Our study proposes a phenotyping tool for the sunflower/*O. cumana* interaction under controlled conditions through image analysis for broomrape tubercle analysis at early stages of the interaction.

**Results:**

We optimized the phenotyping of sunflower/*O. cumana* interactions by using rhizotrons (transparent Plexiglas boxes) in a growth chamber to control culture conditions and *Orobanche* inoculum. We used a Raspberry Pi computer with a picamera for acquiring images of inoculated sunflower roots 3 weeks post inoculation. We set up a macro using ImageJ free software for the automatic counting of the number of tubercles. This phenotyping tool was named RhizOSun. We evaluated five sunflower genotypes inoculated with two *O. cumana* races and showed that automatic counting of the number of tubercles using RhizOSun was highly correlated with manual time-consuming counting and could be efficiently used for screening sunflower genotypes at the tubercle stage.

**Conclusion:**

This method is rapid, accurate and low-cost. It allows rapid imaging of numerous rhizotrons over time, and it enables image tracking of all the data with time kinetics. This paves the way toward automatization of phenotyping in rhizotrons that could be used for other root phenotyping, such as symbiotic nodules on legumes.

**Supplementary Information:**

The online version contains supplementary material available at 10.1186/s13007-021-00779-6.

## Introduction

Sunflower (*Helianthus annuus)* is one of the most important crops for oil production worldwide, and sunflower-cultivated areas have increased in recent decades [1], notably due to the use of hybrid varieties with improved yield, oil content and oil quality. The main area for sunflower is the European continent, where one of the main biological threats is the obligatory parasitic plant *Orobanche cumana* (sunflower broomrape). Concomitantly with the increase in sunflower-cultivated areas, the number of *O. cumana*-infested fields has also increased, particularly in Eastern Europe, Russia and Spain, and can lead to 100% loss depending on sunflower resistance.

*O. cumana* is an obligate parasitic organism of sunflower (*H. annuus*). Its developmental cycle can be divided into four main stages [2]. In the vicinity of sunflower roots, *O. cumana* seeds germinate when perceiving molecules from sunflower root exudates, including strigolactones, such as heliolactone [3], and sesquiterpene lactones, such as dehydrocostus-lactone [4]. Following germination, the germinating tube forms a specific parasitic organ called the haustorium, which attaches and penetrates inside the host root to connect to the vascular system of the sunflower roots. The broomrape takes up water and nutrients from the host and forms storage organs, called tubercles, each of which subsequently develops a single flowering shoot that emerges above ground, forming thousands of tiny seeds within a few months. By parasitizing sunflower plant nutrients for their own benefit, *O. cumana* is very detrimental to sunflower plant and seed development.

Genetic resistance is one of the most powerful control methods. The first sunflower resistance genes were named *Or1* to *Or5* for resistance to races A to E, respectively [5]. However, as a consequence of selective pressure, monogenetic resistance was overcome by new more virulent *O. cumana* races, named races F to H [6, 7]. Various research studies have identified several major quantitative trait loci (QTL) for these new virulent *O. cumana* races [2, 8–11]. These QTL are distributed throughout the sunflower genome, can be dominant or recessive, race specific or nonrace specific and provide different levels of quantitative resistance. Some resistance genes act on the early stages of the interaction, such as incompatible attachment [12] or tubercle necrosis [2]. Posthaustorial/secondary resistances affect later stages of the interaction as the *Orobanche* shoot develops [13]. Although no information is available on most resistance genes, *HaOr7,* a race F resistance gene, has recently been identified and encodes a leucine-rich-repeat receptor-like kinase [12]. Together with the need for new resistance genes to the most virulent *O. cumana* races, a better understanding and definition of the various races are also needed [6]. Finally, to improve resistance durability, it is important to characterize the resistance mechanisms controlled by the various QTL or major genes identified to combine several mechanisms acting at various stages of the parasitic plant life cycle. Therefore, pyramidal resistance would be more difficult to overcome by broomrape populations. The construction of pyramidal resistance will require the screening of more sunflower genetic resources, including wild *Helianthus* species. Indeed, Seiler and Jan [14] have shown the abundance of broomrape resistance to the most virulent races of *O. cumana* in wild *Helianthus* species*.* Wild *Helianthus* species are more complex than *Helianthus annuus* (annual, 2n = 34 and 3.6 Gb) because they can be annual or perennial and because they differ in their genome size and ploidy [15]. While most perennial *Helianthus* species are immune to broomrape, resistance to the most virulent *O. cumana* races is scarcer in annual *Helianthus* species [14]. Resistance genes are introgressed into sunflower varieties through interspecific hybridization and recurrent selection. Since the first resistance genes were developed as early as the 1910s in Russia, derived from interspecific crosses with *H. tuberosus*, other resistance genes have been introgressed into sunflower from *H. deserticola* [16] and *H. debilis* ssp. *tardiflorus* [17], and numerous interspecific hybrids have been evaluated for their resistance [18]. Wild *Helianthus* species were originally collected in North America by the United States Department of Agriculture (USDA) and are maintained in major gene banks [19].

Phenotyping screens for new resistance to *O. cumana* have been mostly performed in infested fields at the latest stage of the interaction by recording the number of broomrape emergences on sunflower plants. The disadvantages of sunflower resistance evaluation in the field are the heterogeneity of the inoculum (*O. cumana* seed content) in the soil, the possible variability over time, the needed time (several months) and the lack of information about the resistance mechanisms involved during the earliest stages [20]. Other approaches for phenotyping the interaction have been proposed, relying on indirect measures of broomrape attack, through leaf chlorophyll or fluorescence measures. Hyperspectral measurements such as red/far-red measures [21], blue green fluorescence and thermal imaging [22], far-near infrared and shortwave infrared reflectance values [23], and plant height and first internode length measured by 3D imaging [24] have been shown to discriminate between broomrape-infected plants and healthy plants. This discrimination appeared from two weeks [21, 22] to 31 days after inoculation [23] and 750 growing degree days [24]. However, these approaches relying on indirect measurements of infestation do not provide information on the stage of interaction affected in the case of resistance and could be affected by the vegetative architecture and growth of sunflowers depending on the genotype.

Phenotyping screens performed at various stages of the interaction would provide information on the stages affected by the resistance and will help in identifying various types of resistance. In addition, compared to field conditions, the use of controlled conditions and the reduction of the screen duration are major parameters for screen optimization. For this purpose, Labrousse et al. [25] developed an early-stage phenotyping screen in Petri dishes that was modified by Louarn et al. [2] and used to map QTL. Using rhizotrons (transparent Plexiglas boxes), this method enables the monitoring of broomrapes on sunflower roots for a few weeks and allows the observation of early stages such as compatible/incompatible attachments, tubercle development and tubercle necrosis.

However, the throughput of such experiments is limited. A first step toward an increase in the throughput is to optimize data acquisition and analysis. Optimization has been performed for root system architecture phenotyping, and numerous tools have been developed in recent years to automate culture, such as the Growscreen platform developed for *Arabidopsis* root and shoot phenotyping [26, 27], as well as imaging developments based on image analysis [28] or X-ray tomography [29]. However, only a few automated systems are available to our knowledge for phenotyping plant interactions with microorganisms or with other plants. A high-throughput imaging plant root system has been developed using dedicated rhizotubes for legumes (*Medicago truncatula*) and various crops [30]. However, the size of the rhizotubes limits the phenotyping of large plants such as sunflower. For a few years, low-cost Raspberry Pi computers and cameras have been used successfully for phenotyping plant leaves [31] or plant growth [32, 33]. We developed a low-cost phenotyping tool for the sunflower/*Orobanche* interaction at the tubercle stage, and we optimized image acquisition and analysis for broomrape tubercles. We coupled culture in rhizotrons with an image acquisition system for the automatic counting of the number of tubercles using a Raspberry Pi computer and ImageJ freeware. This phenotyping system, named “RhizOSun”, was evaluated using a susceptible genotype (2603) inoculated with a French *O. cumana* race. It was then used to phenotype 5 resistant and susceptible sunflower lines inoculated with two *O. cumana* races. Automatic tubercle counting through image analysis led to the same results as manual phenotyping. Finally, we discuss the advantages, limitations and potential improvements of RhizOSun.

## Results

### Tubercle observation of sunflower plants inoculated with *O. cumana* grown in rhizotrons

**Fig. 1:**
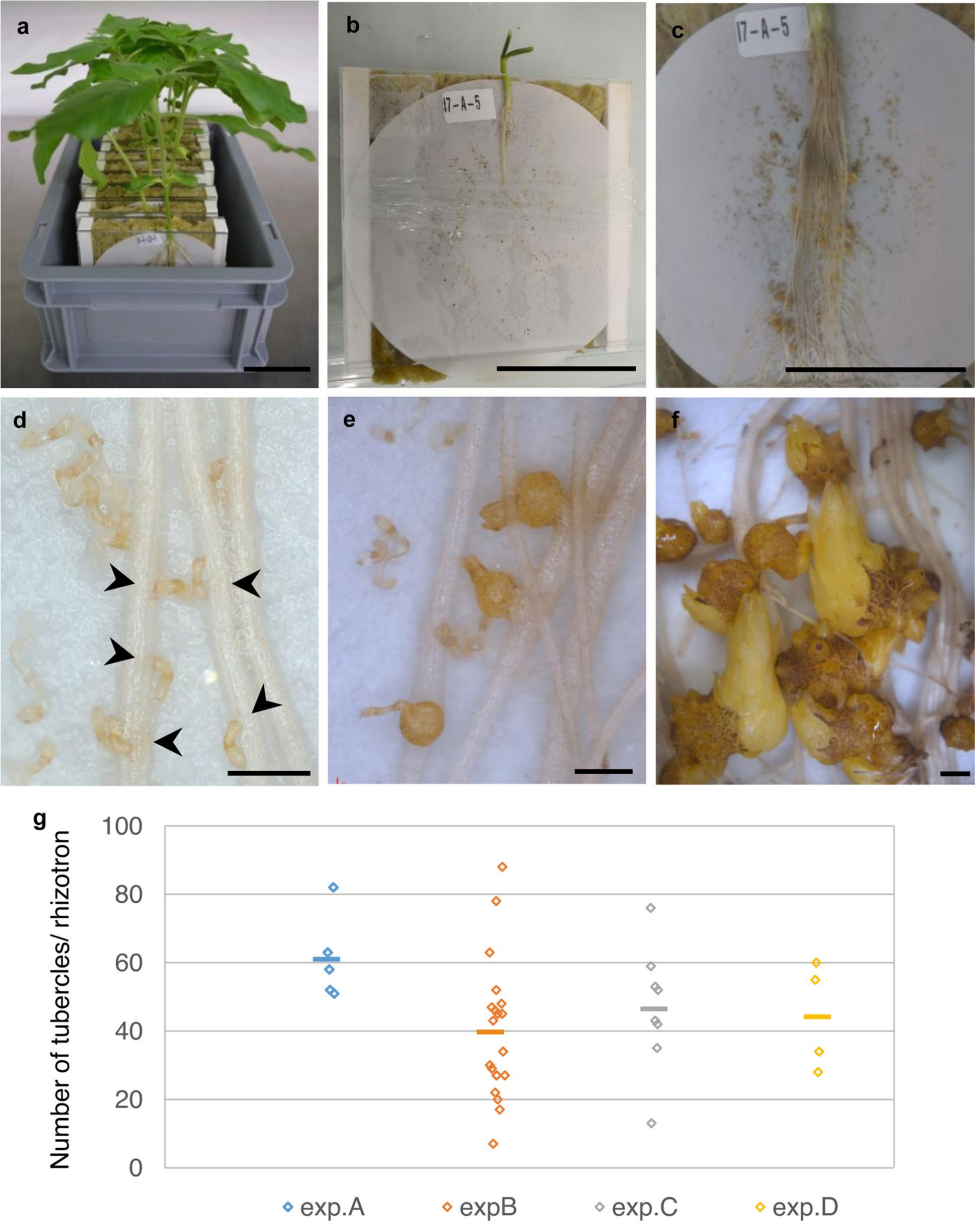
Phenotyping *O. cumana* tubercles on inoculated sunflower roots grown in rhizotrons. The susceptible sunflower genotype (2603) was inoculated with the race Bourret (France) of *O. cumana* in rhizotrons, and the number of tubercles was counted following 3 weeks of culture. **a** Box containing ten rhizotrons at 3 weeks of culture. **b** One-day-old plantlet following rhizotron setup. **c** Inoculated sunflower root system following 3 weeks of culture. **d** First attachments visible following 8 days of culture (arrowheads). **e** First tubercles following 15 days of culture. **f** Buds formed from tubercles following 35 days of culture. Pictures **d–f** were taken under a binocular microscope. **g** Distribution of the number of tubercles/rhizotron for 4 independent experiments at 3 weeks of culture. The mean for each experiment is represented by a bar. **a–c** Bar = 5 cm. **d**, **e** bar = 1 mm. **f** bar = 2 mm

*O. cumana-*inoculated sunflower plantlets were grown in rhizotrons consisting of transparent Plexiglas boxes containing a layer of rockwool and glass fiber paper watered with nutrient solution (Additional file 1: 1a–c, e). In contrast to phenotyping in the field, the use of rhizotrons for growing *O. cumana*-inoculated sunflower allows the observation of early stages of the interaction between the parasitic plant and its host from the germination induction of *O. cumana* seeds to the tubercle stage. Resistance in sunflower accessions can be characterized at the attachment stage (compatible/incompatible [2, 12]) and at the tubercle stage (quantification of the number of tubercles and necrosis of the tubercles [2]). The number of tubercles of inoculated sunflower roots allows us to discriminate susceptible and resistant sunflower genotypes at an early stage. Using sunflower genotype 2603 and *O. cumana* Bourret, the first attachments and first tubercles were visible at 8 days (Fig. 1d) and 15 days (Fig. 1e) post inoculation respectively. The development of buds from tubercles could be observed after one month of culture (Fig. 1f). Variability in the number of tubercles was observed at three weeks of culture among rhizotrons for the same combination of sunflower genotype/broomrape race (Fig. 1g), but the difference in the number of tubercles among experiments was not statistically significant (p = 0.20; 4 independent experiments, a total of 36 rhizotrons).

### Picamera/Raspberry image acquisition apparatus setup and ImageJ macro for automatic counting of the number of tubercles

**Fig. 2:**
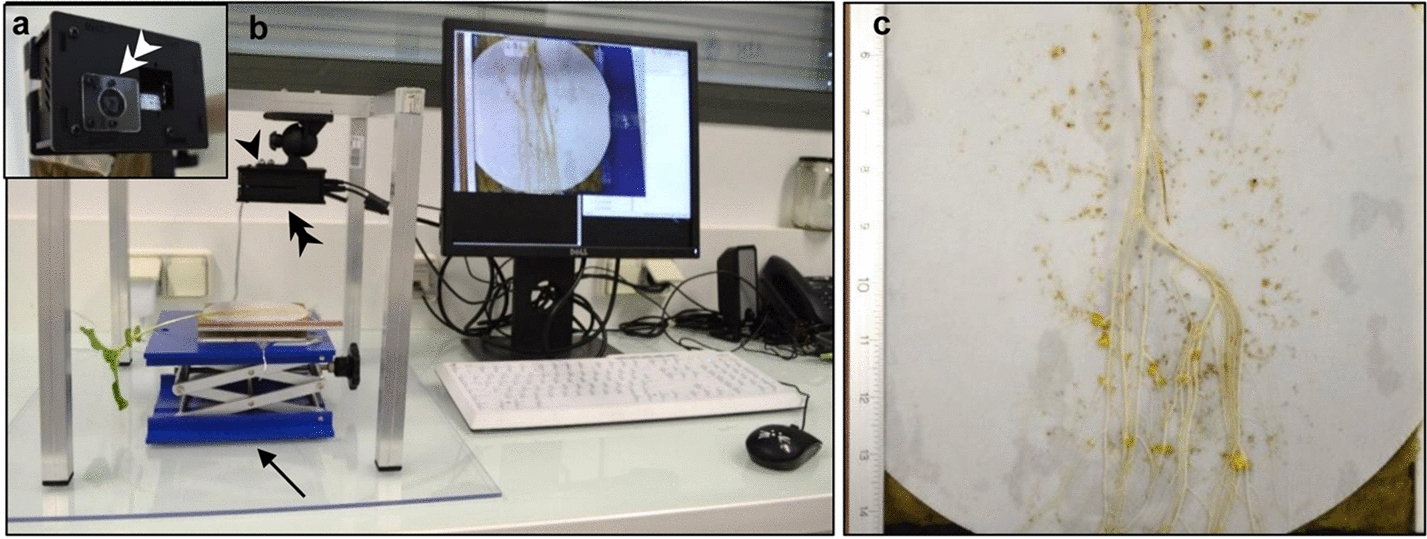
Raspberry Pi/picamera image acquisition setup. **a** Bottom view of Raspberry Pi/picamera (double arrowhead) plugged into the Raspberry computer; the whole set up in a box. **b** Dedicated image acquisition setup of an *O. cumana-*inoculated sunflower root system in a rhizotron using a Raspberry Pi computer protected in a box (black arrowhead), a Raspberry Pi/picamera facing the rhizotron (double arrowhead), connected to a screen, keyboard and mouse. A laboratory lift support (arrow) allows easy focus and field view adjustment for imaging. **c** A picamera image of a rhizotron after 3 weeks, with a ruler on the left for scale adjustment

**Fig. 3:**
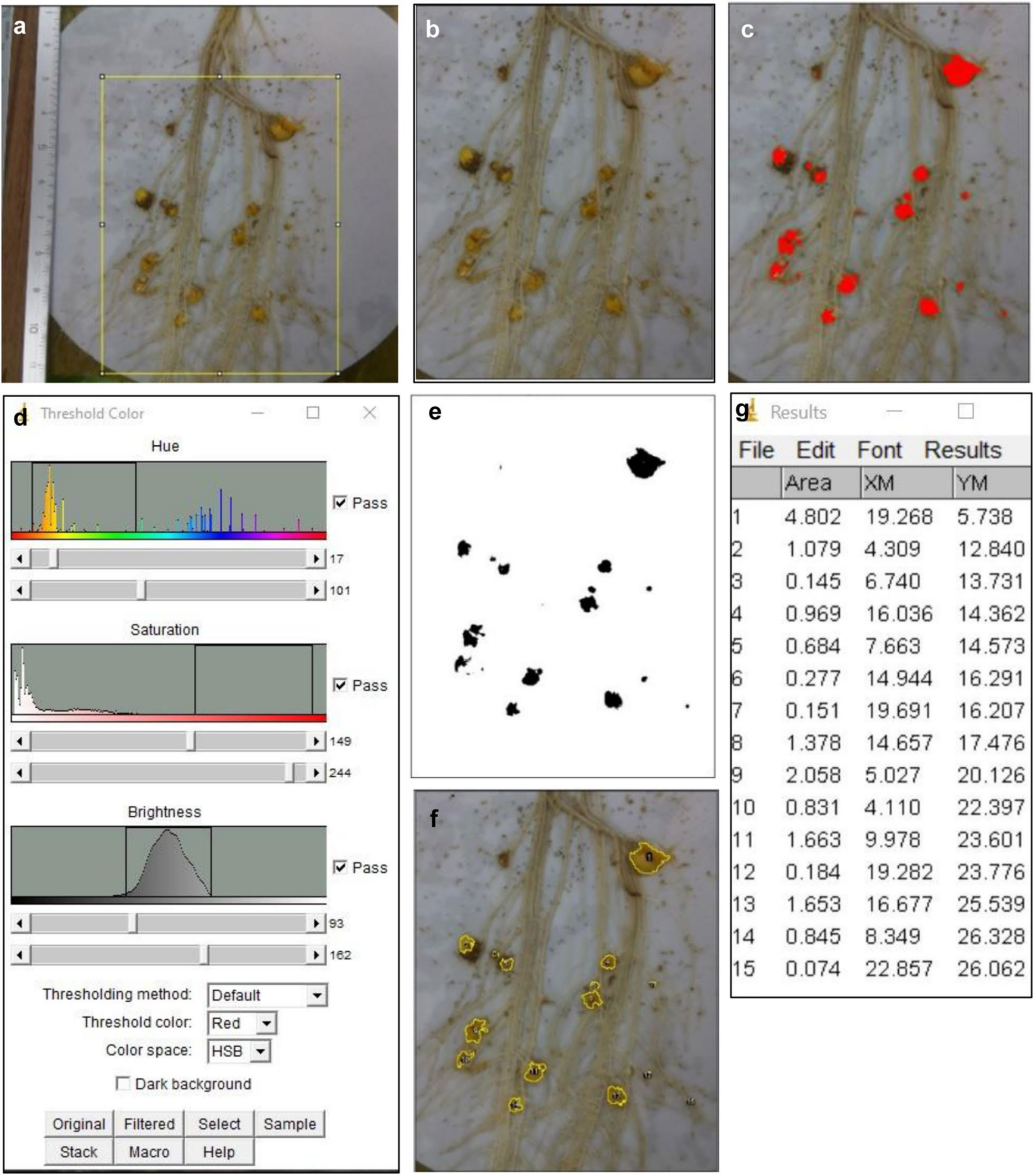
Steps for image analysis for automatic counting of the number of tubercles. To facilitate the visualization of the various image analysis steps toward the identification of tubercles, a 4-week-old inoculated sunflower root was used instead of a 3-week-old root. First, the scale was defined in mm (1100 pixels = 50 mm in this example). Tubercles were identified thanks to the tubercle orange color and defined size. **a** Cropping of the image. **b** The cropped image. **c**, **d** Color threshold window to define HSB parameters (hue = 0–57, saturation = 171–255, brightness = 92–255 in this case) and resulting image (**c**). **e** Binary mask showing the tubercle in black, with filled holes. **f** Original image with drawing of counted particles. **g** Table of the number of particles with their position (center of mass) and area (parameters previously defined in the ImageJ interface: analyze-set measurements)

A picamera plugged into a Raspberry Pi computer acquired images of the inoculated sunflower root system grown in rhizotrons (Fig. 2, Additional file 2). Standardization of images was achieved thanks to the fixed support carrying the camera. The slightly orange color of the tubercles was different from the white color of the sunflower roots and the white filter paper. We counted the number of tubercles by color contrast using a color threshold and a defined size of the particles detected by image analysis. The successive steps of image analysis were illustrated for 4-week-old tubercles (Fig. 3). We defined the scale in mm to facilitate the definition of the size of the particle in mm^2^ rather than in pixels and to avoid any dependence on the image resolution (Fig. 3a). We cropped the image to the region of interest to keep only the inoculated part of the root and reduced the identification of false positive particles (Fig. 3b). Particles were then identified thanks to a manually adjusted definition of a color threshold using hue, saturation and brightness (HSB) parameters (Fig. 3c, d). Once the HSB color threshold was defined, a binary mask was applied (Fig. 3e), and particles were counted (Fig. 3f). The area and center of mass measurements could be preselected to provide additional information on tubercle size and position, respectively (Fig. 3g).

**Fig. 4:**
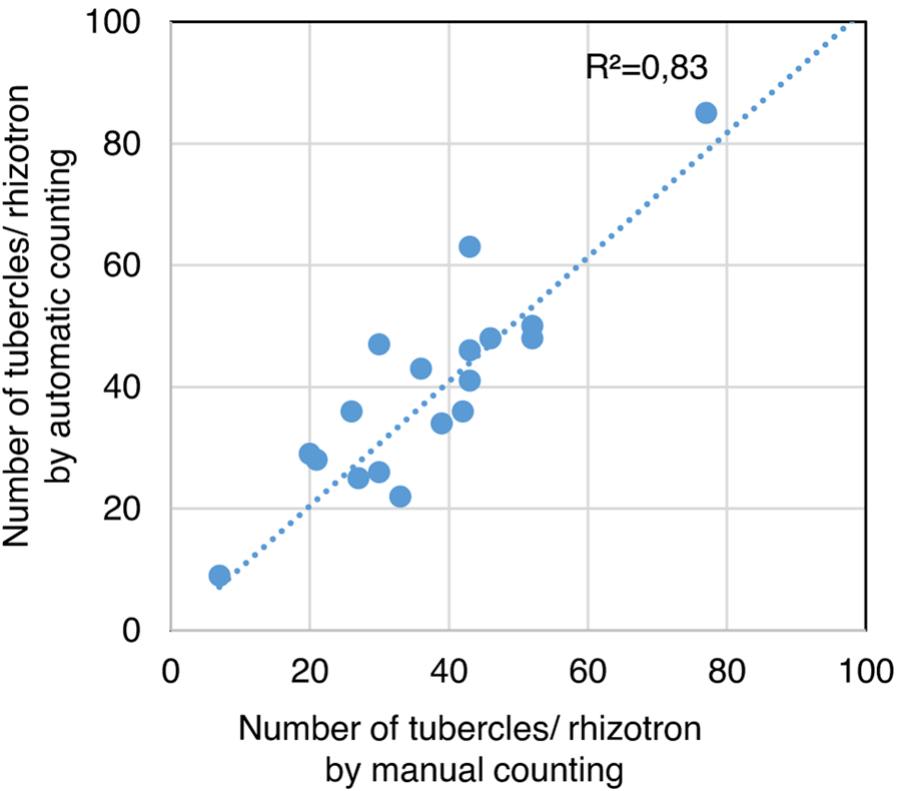
Comparison of the number of tubercles in inoculated sunflower roots using manual counting versus automatic counting. Results for one experiment (19 rhizotrons) using the susceptible 2603 genotype inoculated with the *O. cumana* race Bourret at 3 weeks of culture. Comparison of manual counting (x axis) with automatic counting (y axis) using the ImageJ macro (correlation coefficient = 0.83). (The values defined in the macro are as follows: hue = 0–55, saturation = 102–255, brightness = 95–255; the size of particles is between 0.05 and 20 mm^2^)

Once scale, crop and color threshold values were defined on a few images, an automatic macro could be used for a serial analysis of many images saved in the same directory. The macro contained a first step for automatic opening of images in a defined directory, with a loop for successive image opening, followed by the various steps of analysis described above, and a final step creating a summary table with the number of tubercles found for each image of the directory (Additional file 3: 3a and Additional file 4). In addition, in separate tables, the size and position of the tubercles for each image were available. This macro was used on a series of 19 images (susceptible sunflower line 2603 inoculated with the French broomrape population from Bourret), and for each image, the number of tubercles was compared with the number obtained by manual counting (using the magic wand tool of ImageJ). Figure 4 illustrates the correlation between manual counting and macrocounting. The correlation coefficient was 0.83 (trend line y = 1.02x). Moreover, by comparing matches between manual counting of tubercles with automatic particle counting for each of the 19 rhizotrons, we found that on average, 88 ± 4% of the manually counted tubercles were also counted by automatic macrocounting, and 83 ± 7% of the automatically counted particles were found manually. Additional tubercles (false positives) in automatic counting compared to manual counting came from falsely identified tubercles in the rockwool background or at the stem base if the cropping was not accurate enough (Additional file 6: 6a-b) and counting of very small tubercles were forgotten in manual counting (false negatives) (Additional file 6: 6c, d). By contrast, automatic counting assembled very close tubercles into one particle (Additional file 6: 6e, f) and did not count pale tubercles because of their lighter color outside of the threshold values (Additional file 6: 6g, h). To increase the accuracy of the automatic detection of the tubercles, we tested a semiautomatic macro in which optional manual corrections could be done. The same steps for the successive image analysis were used, but the scale and crop were defined manually for each image, and the number of tubercles could be corrected manually by deleting false tubercles and adding undetected tubercles before the final counting (Additional file 3: 3b and Additional file 5). However, these additional manual steps were time-consuming. The time for counting the number of tubercles of 19 images was 1044 s (55 s/image) for manual counting, 70 s (3.7 s/image) for automatic macrocounting and 550 s (28.4 s/image) for semiautomatic macrocounting. Automatic counting was approximately 15 times more rapid than manual counting. Although semiautomatic macrocounting improved the accuracy of tubercle detection, it was only 2 times faster than manual counting.

### Phenotyping of contrasting sunflower genotypes inoculated with two races of *O. cumana*

**Fig. 5:**
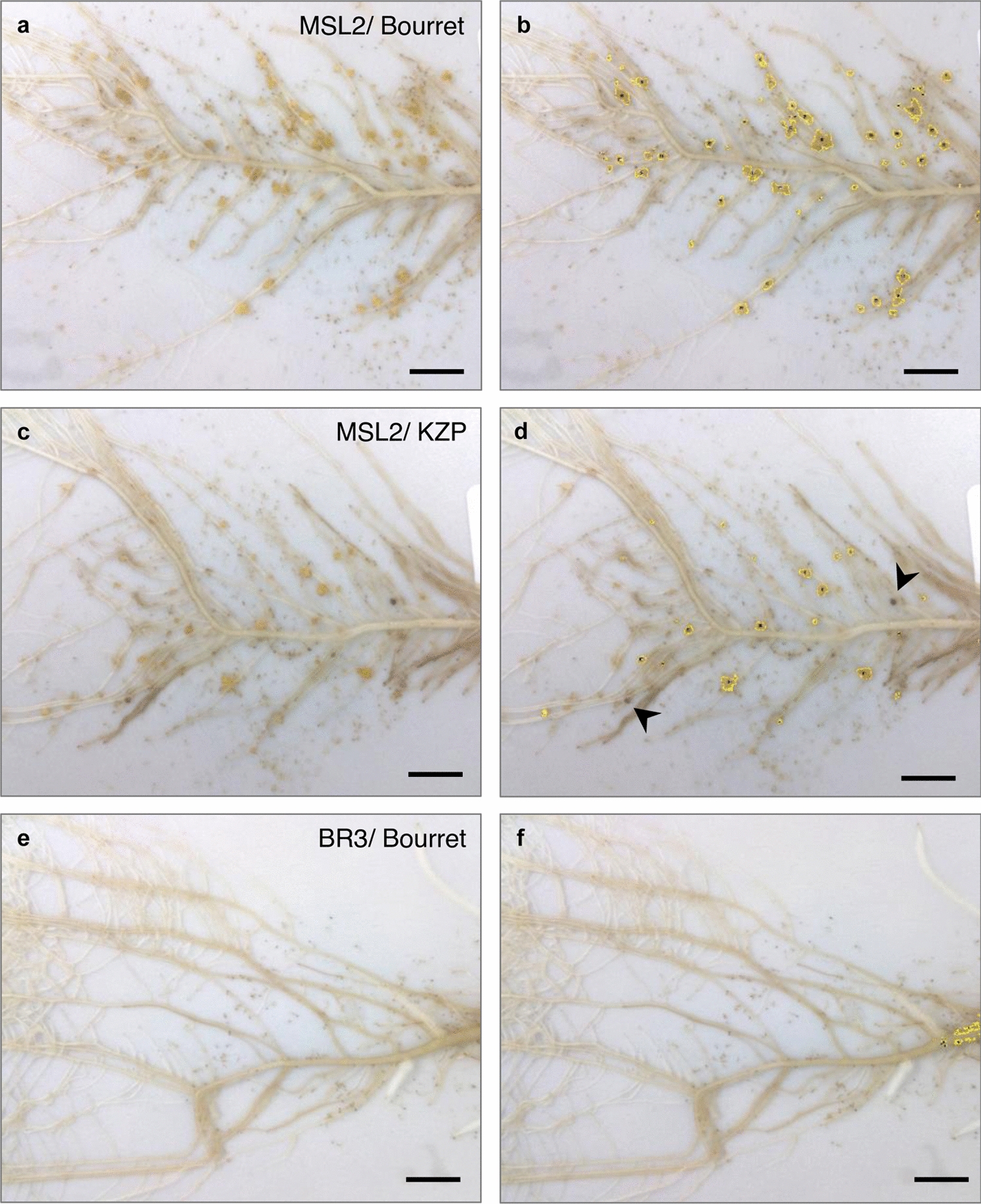
Various susceptibility/resistance phenotypes observed at the tubercle stage using RhizOSun. Phenotyping using RhizOSun: images of 3-week-old inoculated roots (left side, **a-c-e**) and identified particles using automatic image analysis (right side **b-d-f**). **a-b** Susceptible sunflower genotype MSL2 inoculated with the *O. cumana* race Bourret. **c-d** Tolerant sunflower genotype MSL2 inoculated with race KZP: low number of tubercles and development of necrotic tubercles. Necrotic tubercles (arrowheads) were not counted automatically, as shown in **d**, due to their brown color. **e–f** Resistant sunflower genotype BR3 inoculated with the race Bourret (no tubercles). Bar = 1 cm

We phenotyped 5 sunflower genotypes inoculated with two races of *O. cumana.* The sunflower genotypes were chosen because of their different levels of resistance from previous screens. The various phenotypes observed are illustrated in Fig. 5: susceptibility leading to the development of tubercles (Fig. 5a, b), appearance of late resistance and necrotic tubercles (Fig. 5c, d), and complete resistance with no tubercle development (Fig. 5e, f). More detailed phenotypes of each sunflower genotype inoculated with the two *O. cumana* races are illustrated in Additional file 7 and Additional file 8. The sunflower genotype 2603 was susceptible to both *O. cumana* populations developing a high number of tubercles (average of 53 tubercles/ rhizotron). The sunflower genotype BR3 was resistant to the Bourret *O. cumana* population (no tubercles) and susceptible to the KZP broomrape population (presence of tubercles). The LC1093 sunflower genotype was resistant to the Bourret population and tolerant to the KZP population (average < 5 tubercles/ rhizotron). The MSL1 sunflower genotype was resistant to both *O. cumana* races. The MSL2 sunflower genotype was susceptible to the Bourret population and showed a posthaustorial resistance phenotype to the KZP population with necrotic tubercles.

**Fig. 6:**
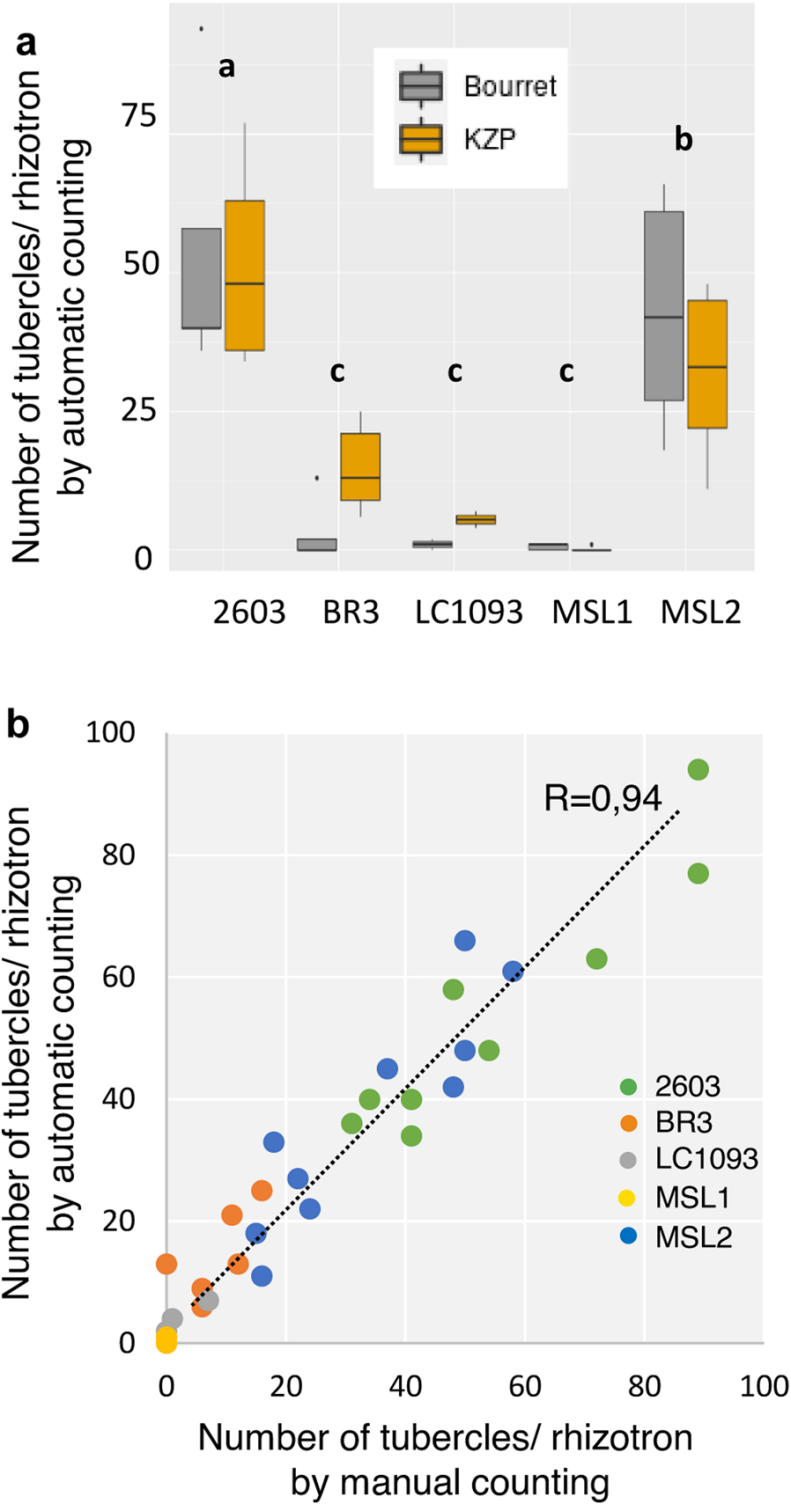
Use of RhizOSun with 5 sunflower genotypes and 2 races of *O. cumana.* **a** Distribution of the number of tubercles/rhizotron (by automatic counting) for each genotype (2 to 5 rhizotrons)/race, Bourret shown as gray bars and KZP shown as orange bars. The letters a-b-c refer to three groups of genotypes that differ significantly (two-way ANOVA: p < 0.001). **b** Correlation between manual counting (x axis) and automatic counting (y axis, R^2^ = 0.94) of the number of tubercles (each genotype is represented in a different color: 2603 = green, BR3 = orange, LC1093 = gray, MSL1 = yellow, MSL2 = blue). The macroparameters HSB and size of particles are similar to the macro used in Fig. 4. The crop size was reduced (compared to the macro used in Fig. 4) to adapt to this batch of images

We used the same automatic macro as in Fig. 4 to characterize tubercle development on the 5 sunflower genotypes inoculated with the 2 races of *O. cumana*. The HSB values were identical for the 5 sunflower genotypes and 2 *O. cumana* races and the same as those used previously. The only modification in the macro was the reduction of the cropping area to limit false particle detection. The distribution of the number of tubercles for each genotype/race condition, counted automatically, is illustrated in Fig. 6a. In this experiment, the sunflower genotypes were divided into 3 groups with significant differences at the statistical level (p < 0.001): one group was the susceptible control genotype 2603, an intermediate group comprising MSL2, and the 3^rd^ group was the most resistant genotypes BR3, LC1093 and MSL1. No significant differences at the statistical level were found among the 2 *Orobanche* races (p = 0.90). These statistical results were similar to those obtained using manual counting of the number of tubercles (effect of the genotype: p < 0.001; effect of the race: p = 0.56). Figure 6b illustrates the values obtained by manual and automatic counts for each genotype. The correlation between manual counting and automatic macrocounting was high (0.94, trend line y = 1.01x). The time needed for automatic counting of the number of tubercles was 4 s/image (the time to place the plate on the support and to acquire the image was not included). The correlation coefficient per sunflower genotype varied from 0.69 to 0.86 (no correlation coefficient could be calculated for genotype MSL1, for which there were no tubercles). We identified more false positive tubercles (Additional file 6: 6b) in the sunflower genotype BR3, explaining the lower correlation coefficient of 0.69. In such cases, manual correction for a few images would be needed to obtain more accurate counting. Considering the *O. cumana* races, the correlation coefficients were 0.97 and 0.93 for the Bourret and KZP populations, respectively.

## Discussion

### Effect of culture conditions on the interaction

In this study, we present an efficient tool, which we call RhizOSun, for phenotyping sunflower roots inoculated with the parasitic plant *O. cumana* at the tubercle stage. This system assembles cultures in rhizotrons, acquires root images and automatically analyzes images of the number of tubercles. We used the culture technique in rhizotrons developed by Louarn et al. [2] with a slightly modified nutrient solution; the phosphate concentration in the half-strength Long Ahston nutrient solution was increased from 7.5 to 732 µM. The increase in the phosphate concentration did not prevent *O. cumana* seed germination and consequently tubercle development but resulted in a higher number of tubercles in a sunflower root system. We observed an average of 44.6 ± 6.3 tubercles/sunflower plantlets at 3 weeks of culture compared to approximately 12 tubercles/sunflower reported by Louarn et al. [2] at 5 weeks of culture using the same susceptible genotype 2603 and similar growth conditions. The nutrient solution composition was shown to impact the distribution of *O. cumana* seed germination stimulants in sunflower root exudates [3] as well as in tobacco root exudates [34]. Sunflower root exudates contain a cocktail of germination stimulants, some of which are potentially susceptible to phosphate starvation, such as heliolactone [3], and others for which the concentration is independent of phosphate concentration, such as dihydrocostus lactone [4], as suggested by our results. Increasing the phosphate concentration in the nutrient solution allowed us to keep the culture for a longer time if necessary, as the plants did not suffer from starvation, and emergence of *O. cumana* could eventually develop after 6 weeks of culture. The variability in the number of tubercles among rhizotrons for the same combination of sunflower genotype*/O. cumana* race, even though no significant difference was observed among experiments, remains a problem for comparing slight differences among genotypes. This problem could be overcome by increasing the number of rhizotrons/ genotype as well as the use of a standard susceptible control included in each independent experiment. In addition, this variability does not prevent the discrimination of resistant sunflower plants from susceptible ones. We did not measure sunflower root length or growth. However, we identified plants showing an obvious reduction in root development without a major effect on the number of tubercles. It would be interesting to characterize the effect of root development on the interaction together with the locations of the broomrapes on the root system. Culture in rhizotrons is an efficient tool for such objectives. Because the method is not destructive, observations can be performed throughout the culture to monitor the interaction from *O. cumana* seed germination to attachments or tubercle development, allowing kinetic analysis.

Using rhizotron culture enables to control and study environmental parameters in the *Orobanche*/sunflower interaction. Indeed, temperature has been shown to increase the degeneration of tubercles of the resistant sunflower variety Ambar [35] and, by contrast, increase *Orobanche* development in the susceptible sunflower variety Adi using inoculated sunflowers grown in polyethylene bags in phytotrons. Understanding the effect of temperature on the interaction mechanisms will be of particular interest in the context of climate change. Moreover, using rhizotrons watered with various nutrient solutions (water, half-strength and full-strength Coïc solution), Labrousse et al. [36] showed that nutrient concentrations can affect the phenotype of resistant and susceptible genotypes during the sunflower*/O. cumana* interaction. Our RhizOSun system will facilitate future studies on the effect of various physical and chemical parameters on the interaction between sunflower and *Orobanche cumana* at early stages*.*

### Image acquisition and analysis

RhizOSun took advantage of the low-cost image acquisition Raspberry Pi/picamera system. Using a Raspberry Pi/picamera system allowed us to set up a low-cost dedicated image acquisition apparatus for rhizotron imaging for sunflower*/O. cumana* research studies in a confined laboratory environment required for experiments with this parasitic plant to prevent dissemination. It also allowed us to standardize the image acquisition procedure (image focus and field of view) to facilitate image analysis, thanks to the dedicated rhizotron laboratory lift support. In the context of sunflower/*Orobanche* interactions, the images obtained by the picamera were of sufficient definition and quality to enable image analysis and count the number of tubercles. Using image acquisition of all inoculated root systems represents a great advantage over manual counting, as it allows archiving of all data for additional analyses, as well as image analysis shifted in time if necessary, relaxing the experimental organization.

We chose ImageJ software for image analysis, as it is one of the most commonly used software programs for scientific image analysis, in addition to being free and open source, thus facilitating the transfer of this method to other laboratories. Image analysis of the tubercle could be realized thanks to the natural orange color of the tubercle, discriminating tubercles from white-colored roots and the white glass-fiber paper used as a background. Choosing appropriate color threshold values for hue, saturation and brightness on a few representative images needed to be set up at the beginning of the experiment. However, once these values were validated, the gain of time for image analysis was very high, as manual counting required almost 1 min/image compared to approximately 4 s/image using the automatic ImageJ macro, which was 15 times faster. Interestingly, 88 ± 4% of tubercles were recognized by automatic counting. Optional manual corrections based on scale, cropping and the number of tubercles increased the correlation between manual counting and semiautomatic counting, but by using all these options, the gain of time was only half (approximately 30 s/image). However, each option could be selected independently by users who can modify each step of the macro. Manual correction of the number of tubercles was particularly valuable for completely resistant genotypes, which could appear as tolerant with a low number of particles (< 10/ rhizotron) by automatic counting, to delete false positive particles, and this can be done subsequently for the most interesting genotypes. Once evaluated on the interaction between the susceptible genotype 2603 inoculated with the race Bourret, we used RhizOSun to phenotype four additional sunflower genotypes inoculated with two *O. cumana* races. RhizOSun phenotyping led to an excellent correlation (R^2^ = 0.94) of the numbers of tubercles compared to manual counting, despite the use of various genetic resources, showing that tubercle color was sufficiently uniform among sunflower genotypes and *O. cumana* races to be used for particle identification with the HSB settings previously defined for the susceptible genotype 2603. In our program, the necrotic tubercles, because of their brown color, were not counted because they were not recognized. If necessary, a second counting with adequate hue, saturation, and brightness values for these brown-colored tubercles could be added. Moreover, other measurements could be performed automatically if required, such as the total area and positions of tubercles, adding other information on the development of tubercles. Finally, as machine learning is increasingly applied in plant science [37], it can be used thanks to the bank of images acquired by RhizOSun. This could lead to the improvement of the automatization, to correctly separate groups of close tubercles, and to count tubercles of various colors.

### Comparison of RhizOSun with other phenotyping tools

The RhizOSun system allows the phenotyping of sunflower*/O. cumana* within one month of culture at the tubercle stage compared to 3–4 months for phenotyping at the emergence stage in pots or in the field. In addition, phenotyping under controlled conditions is possible throughout the year, in contrast to once a year for field phenotyping. The density of rhizotrons was 166 plants/m^2^ under our conditions, meaning that 1992 plants/m^2^ could be screened per year. However, even though the RhizOSun system allows information on resistance at early stages of the interaction, it has not been tested for phenotyping late stages of resistance, such as posthaustorial resistance [13], affecting emergence development. Therefore, phenotyping with RhizOSun and phenotyping in pots/fields are complementary approaches. Other phenotyping tools mentioned previously rely on indirect measurements of *O. cumana* development through hyperspectral measures (red/far-red measures [21], blue green fluorescence and thermal imaging [22], far-near infrared and shortwave infrared reflectance values [23]) as well as plant height and first internode length measured by 3D imaging [24]. Although these tools have the potential to be used for the detection of *Orobanche* infestation in fields cultivated with homogeneous hybrid lines, they are not optimized for large screenings of resistant genotypes, as they require twice as many pots for each genotype to compare inoculated plants from control noninoculated plants. In addition, screening nonfixed accessions such as populations or wild *Helianthus* accessions with intragenetic diversity will make such approaches difficult.

## Conclusion and perspectives

In this work, we propose a new tool for phenotyping sunflower resistance to *O. cumana* at early stages, saving time and space compared to field screens and saving time in terms of tubercle counting (4 s image analysis compared to 1 min by manual counting). This low-cost and dedicated apparatus allows archiving images/data. The RhizOSun system will be very useful to screen wild *Helianthus* species for new resistance to the most virulent races of *O. cumana.* This type of screening is currently being developed and requires specific adaptation for these wild species with slow growth compared to the cultivated lines of *H. annuus*. In addition to the interaction between sunflower and *O. cumana,* the Raspberry Pi/picamera and associated macro image analysis based on color threshold have other potential uses in the area of plant–microbe interactions such as screening for symbiotic legume mutants based on the counting of stained nodules. In that case, it is noteworthy that the use of a camera of better quality than the picamera or a camera equipped with a zoom would allow imaging smaller objects than tubercles, such as nodules. Moreover, in line with GROWSCREEN-Rhizo developed for the small-sized model plant *Arabidopsis thaliana* by Nagel et al. [27], the RhizOSun system paves the way for a high-throughput phenotyping automatic platform by automatization of bar-coded rhizotrons, controlling watering, imaging and image analysis. Finally, this work could contribute to a bank of images of tubercles, which would be the basis of expert data for training machine learning models toward improved automatization of tubercle detection on imaged *O. cumana-*inoculated roots of sunflower.

## Materials and methods

### Plant materials

Five sunflower lines were used in this study, with various resistant phenotypes: a susceptible control line 2603 from INRAE, 2 public lines: BR3 [38] and LC1093 [25], and 2 private lines from MAS Seeds: MSL1 and MSL2. Two *O. cumana* races were used: “Bourret” harvested in 2015 (Tarn, France), classified between E and F, and “KZP” harvested in 2016 (Zaporojie, Ukraine), classified as F-G.

### Growth conditions

Plants were grown in rhizotrons as illustrated in Fig. 1a–c and Additional file 1: 1. Sunflower seeds were decontaminated for 10 min with 4.8% sodium hypochlorite, rinsed three times with sterile water, and allowed to germinate in Petri dishes containing a few ml of water in the dark at 25 °C for 3 days. Germinated seedlings were transferred to a mixture 1/1 (v/v) of sand/vermiculite for another 3 days to allow cotyledon development in a growth chamber (22 °C, 60% humidity, 16 h light 110 µE/m^2^/s). In parallel, *O. cumana* seeds were decontaminated for 5 min with 3.2% sodium hypochlorite and 0.001% Triton X-100, rinsed three times with sterile water using a 40 µ sterile cell strainer, and subsequently conditioned in water for 6 days at 22 °C (3.3 mg/ml) in the dark in a 50 ml sterile tube. Rhizotrons, consisting of 2 Plexiglas plates 12 × 12 cm separated by Plexiglas separators 1 cm thick, were assembled with autoclaved precut and water-soaked rockwool (Grodan Expert, Grodan (Rockwool B. V) P.O. Box 1160, 6040 KD Roermond, The Netherlands) and sterile glass fiber paper (reference 036294B, Dutscher). Three-day-old seedlings were transferred to the glass fiber paper and inoculated with 3 ml of conditioned *O. cumana* seeds (10 mg final/rhizotron), and rhizotrons were placed vertically in a growth chamber at 22 °C with 60% humidity (16 h day, 110 µE/m^2^/s). Rhizotrons were watered 3 times per week with half-strength Long Ashton nutrient solution [39] containing 732 µM phosphate, allowing the rockwool to dry between two watering periods. The culture in the rhizotrons was derived from the protocol of Louarn et al. [2], except that the phosphate concentration was increased from 7.5 µM to 732 µM.

### Experimental design

Four independent experiments, comprising 5 (exp. A), 19 (exp. B), 8 (exp. C) and 4 (exp. D) rhizotrons with sunflower genotype 2603 inoculated with the *O. cumana* Bourret population, were used to count the number of tubercles at 21 days in each rhizotron (Fig. 1g). Nineteen rhizotrons (exp. B) were used to test the macro in Fig. 4. One experiment (exp. E) was performed using the 5 sunflower genotypes detailed above, inoculated with the two broomrape races Bourret and KZP, with 5 rhizotrons per sunflower genotype/*O. cumana* race combination, except for the sunflower genotype LC1093 for which there were 2 rhizotrons for each race (because of germination problems with this sunflower genotype).

### Raspberry Pi/picamera setup for image acquisition

We used the Raspberry Pi 2 model B, with a microSD of 16 Gb. It was coupled to a picamera model V2 and completed with a power supply, an SD card reader and a wireless adaptor. The elements were assembled in a picamera box bundle-B + /2/3. References of the various elements are listed in Additional file 2: 2a, and the setup is shown in Fig. 2a, b. The file Raspberry Pi OS (32 bit) with desktop and recommended software (previously named Raspbian-Stretch) was found on the official Raspberry Pi site (https://www.raspberrypi.org/) and was flashed on the micro SD card using Etcher software (balena.io/etcher). Once the file was installed on the micro SD card, it was inserted in the dedicated SD port on the Raspberry Pi as well as the other peripherals (computer screen via HDMI connection, mouse, key-board and power supply lastly). The picamera was connected via the dedicated port. The Raspberry Pi was then started, as the desk interface appeared on the screen, and the Raspberry Pi was connected to the internet via a Wi-fi USB key for the necessary installations and updates. In the preferences/interface, the Secure Schell (SSH) was activated to enable a secure distant connection, the camera was activated, and the country and time zone were defined for internet synchronization and automatic update of time and date. The final command for image acquisition written in the terminal window was “raspistill-o name%03d.tiff –t 0 –p 0,5 –k” (Additional file 1: 2b). This allowed an unlimited time of preview before each image acquisition. Images were acquired by pressing the enter key on the keyboard, and file name was automatically incremented. Once all the images were acquired, the system was closed by pressing the x key on the keyboard, and images could be transferred for analysis via a USB key. Image focusing was first performed by manually turning the lens of the camera, and second, easy image focusing was adjusted by the laboratory lift support. The resolution of the images taken by the picamera was 3280 × 2464 pixels = 8 million pixels.

### ImageJ macro-mediated image analysis

For analysis, images were transferred from the Raspberry Pi computer to a standard computer with a USB key, and ImageJ software [40], version 1.52a, was used. Analysis of the number of tubercles was performed taking into account the natural orange color of tubercles, which allowed their discrimination from white roots and the white filter paper. In brief, 5 successive steps were performed (Fig. 3): (i) set a scale, using a ruler, in the image and define the unit as mm, (ii) crop the images by defining the region of interest (the part of the image with inoculated roots), (iii) isolate particles thanks to a color threshold defined on hue, saturation and brightness values corresponding to tubercle color, (iv) convert particles with a binary mask and (v) analyze the number of particles according to a defined size range. A macro was then written for automatic image opening and analysis in a defined directory, leading to a summary table of the number of identified particles in each image. Additionally, a semiautomatic version of the macro was written, derived from the previous version, in which manual corrections could be made for the following steps: scaling, cropping, and correcting the number of tubercles (by adding or deleting tubercles). In this semiautomatic macro, each manual correction is announced by a dialog window. We summarized the various steps of the fully automatic macro (Additional file 1: 3a) and of the semiautomatic version (Additional file 1: 3b) with manual corrections for scaling, cropping and adjusting the numbers of tubercles. Both scripts are furnished as Additional file 4 and Additional file 5. To define the scale in the automatic macro script (Additional file 4), the user has to draw a horizontal line of “x” mm along the horizontal ruler captured in one image using the “straight line” selection tool in the ImageJ tool bar. The length in pixels (“LP”) is indicated in the status bar of ImageJ and has to be reported in the macro script, Step 2: Set scale, as the third value of the “make line” command (0,0, “LP”,0). The real value (“x”) as well as the unit (mm) of the drawn line have to be defined in the next line of the macro script “run(“Set Scale…”, “known = x unit = mm”). Concerning hue, saturation and brightness, the user has to first select “HSB” as the “color space” and then move the sliders until the desired objects are highlighted in red (when “red” is selected as the “threshold color”). For help in this setting, the user can select a small region of the particles to be identified and click the “Sample” button to predefine the hue, saturation and brightness values. The six values indicated at the end of the sliders have to be reported in step 4 of the macro script in the min[0], max[0] (for hue values), min[1], max[1] for saturation values, min[2] and max[2] for brightness values. For additional information about these commands, the user can refer to the ImageJ website on “Documentation” and “Menu Commands” (https://imagej.nih.gov/ij/docs/guide/146-Part-V.html#toc-Part-V). The macro defined for Fig. 4 was set up with the following parameters: scale = 1100 pixels, 50 mm; cropping image = (400, 426, 2634, 1824), hue = 0–55, saturation = 102–255, brightness = 95–255. This set of values was manually defined based on a few randomly chosen images using the Image/Adjust/Threshold command in ImageJ opening a dialog window, as shown in Fig. 3d. The defined size of the particles was between 0.05 and 20 mm^2^. For Fig. 6b, the parameters of the macro were the same as for Fig. 4, except for the cropping region, which was reduced to 400, 426, 2258, 1824, to avoid false particles at the stem base.

To establish the macro, an initial experiment was conducted using the susceptible genotype 2603 inoculated with the race Bourret in 19 rhizotrons (exp. B). In a second experiment (exp. E), the macro was tested using the 5 sunflower genotypes described above, inoculated with the 2 *O. cumana* races, with a total of 44 rhizotrons and 5 rhizotrons/condition, except for genotype LC1093, for which 2 rhizotrons/condition were observed.

### Statistical analysis

Data were subjected to statistical analysis using Statgraphics Centurion XV. II professional software (Statpoint technologies Inc.). The normality of residuals was verified by the Kolmogorov–Smirnov test. One-factor ANOVA was used for the statistical analysis of the effect of the experiment on the number of tubercles using the 4 experiments A, B, C and D mentioned in the experimental design paragraph. Two-factor ANOVA was used to analyze the effect of sunflower genotype and *Orobanche* race on the number of tubercles counted automatically using experiment E with 44 rhizotrons in total with the 5 sunflower genotypes inoculated with both *Orobanche* races. A similar two-way ANOVA was performed on the number of tubercles counted manually. The correlation coefficient was calculated using Microsoft Excel (2016).

## Supplementary Information

**Additional file 1.** Rhizotron setup.

**Additional file 2.** Commercial references of the various elements for the setup of a Raspberry Pi/picamera dedicated to rhizotron image acquisition (a) and list of commands for image acquisition (b).

**Additional file 3.** Successive steps of the macro developed with ImageJ for counting the number of tubercles from images of rhizotron-grown sunflower plants inoculated with *O. cumana* at 3 weeks of culture*.* Automatical counting (a) or semiautomatic counting with manual options in the macro (b).

**Additional file 4.** Automatic macro developed for ImageJ counting of the number of tubercles.

**Additional file 5.** Semiautomatic macro developed for ImageJ counting of the number of tubercles.

**Additional file 6.** Differences between manual counting and automatic macrocounting of the number of tubercles.

**Additional file 7.** Phenotyping of 5 sunflower genotypes with 2 *O. cumana* races 21 days post inoculation using RhizOSun.

**Additional file 8.** Distribution of the number of tubercles/ rhizotron for 5 sunflower genotypes inoculated with 2 races of *O. cumana*, data obtained by manual counting.

## Data Availability

The datasets used and/or analyzed in the current study are available from the corresponding authors upon reasonable request.
