# Supplementary material for: Image analysis for the automatic phenotyping of Orobanche cumana tubercles on sunflower roots
Source: Plant Methods. 2021 Jul 21;17:80. doi: 10.1186/s13007-021-00779-6 (PMC8293553; doi:10.1186/s13007-021-00779-6)

Additional Materials

Additional File 1. Rhizotron setup.

Desinfected *O. Cumana* seeds were preconditionned in water in a 50 ml sterile tube at 22°C for 6-7 days in the dark **(a).** Desinfected sunflower seeds were germinated in a small amount of water for 3 days (**b**), transferred to a mixture of sand/vemiculite 1/1 v/v (**c**) for another 3 days, before rhizotron setup (**d**). Rhizotron is a 12 x 12 cm Plexiglas box containing precut autoclaved and water-soaked rockwool and a sterile glass fiber paper. 3 ml of preconditioned *O. cumana* seeds solution corresponding to 10 mg of seeds were pipetted on the filter after transferring the sunflower plantlet. (**e**) shows a 21 day-old rhizotron (genotype 2603, race Bourret).

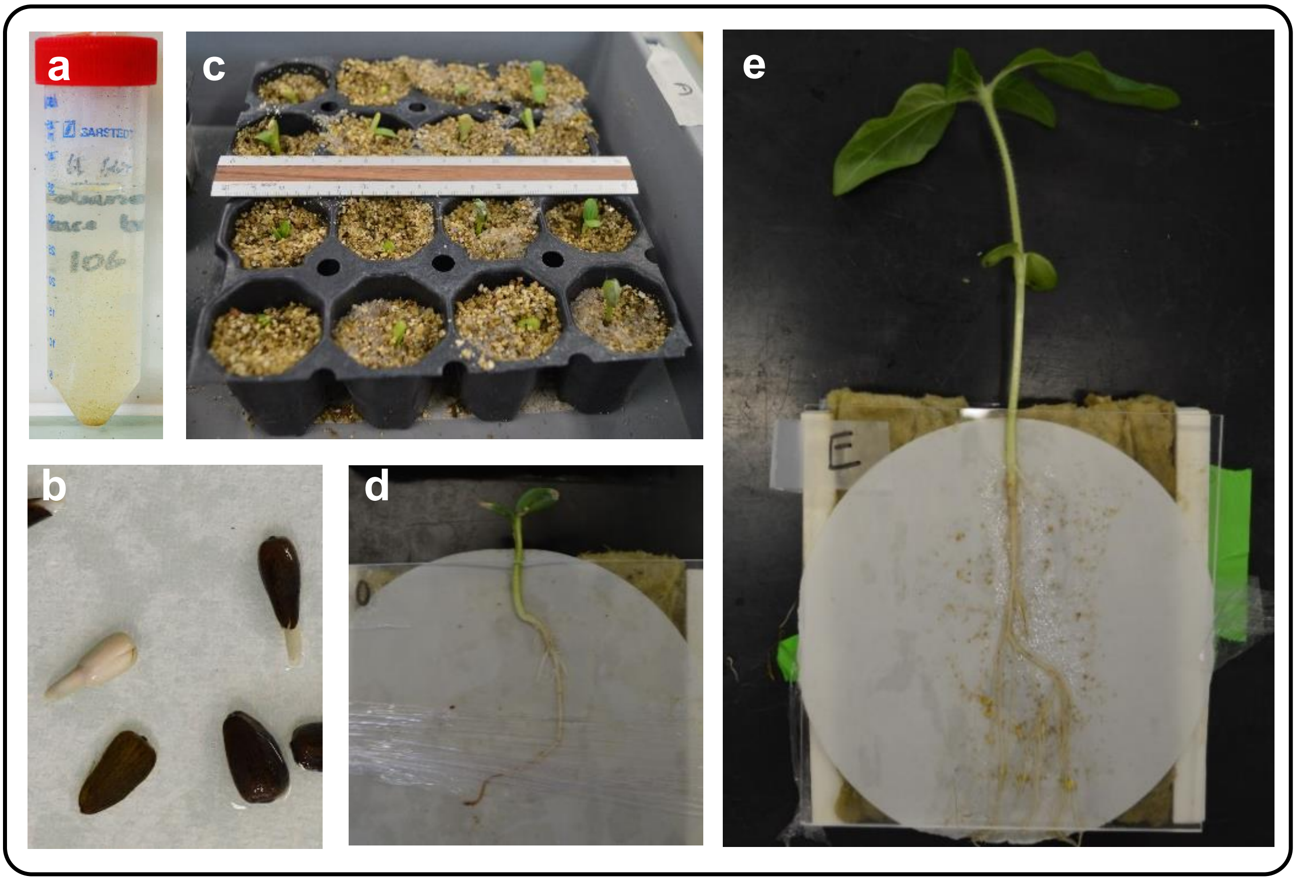

Supplement: Supplementary file 1 — Additional file 1. Rhizotron setup. [file 13007_2021_779_MOESM1_ESM.pdf]
