# Supplementary material for: Image analysis for the automatic phenotyping of Orobanche cumana tubercles on sunflower roots
Source: Plant Methods. 2021 Jul 21;17:80. doi: 10.1186/s13007-021-00779-6 (PMC8293553; doi:10.1186/s13007-021-00779-6)
Supplement: Supplementary file 2 — Additional file 2. Commercial references of the various elements for the setup of a Raspberry Pi/picamera dedicated to rhizotron image acquisition (a) and list of commands for image acquisition (b). [file 13007_2021_779_MOESM2_ESM.pdf]

Additional File 2. Commercial references of the various elements for the setup of a Raspberry Pi/picamera dedicated to rhizotron image acquisition (a) and list of commands for image acquisition (b).

(Lines of code for commands were written in the terminal of the Raspberry Pi and activated by the enter key).

**2a-Elements**	**Reference**	**Provider**
Raspberry Pi 2 model B	8326274	RadioSpares
microSD card Sandisk of 16 Gb	1213897	RadioSpares
picamera model V2	9132664	RadioSpares
power supply	9098126	RadioSpares
SD card reader	7873290	RadioSpares
wireless adaptator	8920012	RadioSpares
Pi camera box bundle-B+/2/3		ModMiPy

In addition a screen, mouse and keyboard and a HDMI/HDMI wire are needed.

2b-Commands for image acquisition

Create a new folder and name it	directory-name
In the terminal, go to the directory	cd directory-name
Initiate commands in the mode of image acquisition	raspistill
Name the image and number them (03 is for 3 digits), save in the tiff format	-o name%03d.tiff
Define unlimited time before image acquisition	-t 0
Preview: allow to see the image directly on the screen (size= 50% of the screen), before taking the picture	-p 0,5
Take the picture, and increment images 1 by 1 thanks to the enter key	-k

The final command is : raspistill –o name%03d.tiff –t 0 –p 0,5 -k

Once the serie of images is finished, press X to end the process, and images can be transfered/recovered on a USB key.
