# Supplementary material for: Image analysis for the automatic phenotyping of Orobanche cumana tubercles on sunflower roots
Source: Plant Methods. 2021 Jul 21;17:80. doi: 10.1186/s13007-021-00779-6 (PMC8293553; doi:10.1186/s13007-021-00779-6)
Supplement: Supplementary file 3 — Additional file 3. Successive steps of the macro developed with ImageJ for counting the number of tubercles from images of rhizotron-grown sunflower plants inoculated with O. cumana at 3 weeks of culture. Automatical counting (a) or semiautomatic counting with manual options in the macro (b). [file 13007_2021_779_MOESM3_ESM.pdf]

**Additional File 3. Successive steps of the macro developed with ImageJ for counting the number of tubercles from images of rhizotron-grown sunflower plants inoculated with *O. cumana* at 3 weeks of culture. Automatical counting (a) or semiautomatic counting with manual options in the macro (b).**

The macro is written using the macro recorder or can be open directly in ImageJ. A directory containing all the images is prepared and the macro is run, starting by opening one image of the directory, and leading to an ultimate table recording the number of tubercles for each image, as a number of particles based on the tubercle orange color of defined size. ImageJ version 1.52a is need.

**3a :** Automatic counting

| **Description of the steps** | **Commands** |
|---|---|
| **Step1 : opening of images, defining the directory path, creating a table recording the number of tubercles for each image and creating a loop for successive image analysis** | |
| Select image to open and get the folder path of the file | path = File.openDialog("Select a File");<br>dir = File.getParent(path); |
| Create a new folder « Results » for the final table of tubercle number<br>Define parameters to be measured in the « Analyze/ Set Measurement » menu (area and center of mass in this case)<br>Create an empty result table to resume the number of tubercles by image<br>Generate the list of the existing files (images to analyze) in the original image directory | newdir=dir+"\\"+"Results";<br>File.makeDirectory(newdir);<br>run("Set Measurements...", "area center display redirect=None decimal=3");<br>Table.create("tubercle number");<br>list = getFileList(dir); |
| Make a loop from the selected directory to open images one after the other and analyze them | for (j=1;j<list.length;j++)<br>{ |
| Clear the previous measurement table before analyzing a new image,<br>Open the next image to be analyzed in the directory with its name | run("Clear Results");<br>open(dir+"\\"+list[j]);<br>name=File.nameWithoutExtension; |
| **Step 2 : set scale** | |
| Set scale<br>As the images are all acquired with the same fixed apparatus so the scale is the same for all images and depend of the equipment. The scale is define in advance on a control image of a ruler. Use the command makeLine (x,y, length, height) and draw | makeLine(0, 0, 774, 0);<br>run("Set Scale...", "known=50 unit=mm"); |

| | |
|---|---|
| a line of 50 mm (in this case) on the ruler. The values in the right column are given as an example and have to be define (plugin/macro/record) on your own image | |
| **Step 3 : crop the region of interest** | |
| Crop the region of interest (region of the root with tubercles) to reduce the size of the analyzed image and take off the borders. This region is define in advance on a control image, depending on the set up and will be the same for all the images. The values in the right column are given as an example and have to be define (plugin/macro/record) on your own image<br>Cropped image is saved in the Results folder with its original name and « _crop » suffix | makeRectangle(400, 426, 2634, 1824);<br>run("Crop");<br>SaveAs("tiff",newdir+"\\"+name+"_crop"); |
| **Step 4 : color threshold and binary mask** | |
| The color threshold value have to be adjusted depending on the color of the tubercles depending on image acquisition light conditions. In this example, the Hue values (min[0] and max[0]) are 0 and 55 respectively; the Saturation values (min[1] and max [1]) are 102 and respectively 255 and the brightness values (min[2] and max [2]) are 95 and 255 respectively. These values are defined empirically, with the HSB mode, on a small number of images, using the command Image/Adjust/Color threshold for the best identification of the tubercles. | min=newArray(3);<br>max=newArray(3);<br>filter=newArray(3);<br>a=getTitle();<br>run("HSB Stack");<br>run("Convert Stack to Images");<br>selectWindow("Hue");<br>rename("0");<br>selectWindow("Saturation");<br>rename("1");<br>selectWindow("Brightness");<br>rename("2");<br>min[0]=0;<br>max[0]=55;<br>filter[0]="pass";<br>min[1]=102;<br>max[1]=255;<br>filter[1]="pass";<br>min[2]=95;<br>max[2]=255;<br>filter[2]="pass";<br>for (i=0;i<3;i++){<br>selectWindow(""+i);<br>setThreshold(min[i], max[i]);<br>run("Convert to Mask");<br>if (filter[i]=="stop")  run("Invert");<br>}<br>imageCalculator("AND create", "0","1");<br>imageCalculator("AND create", "Result of 0","2");<br>for (i=0;i<3;i++){ |

| | |
|---|---|
| | selectWindow(""+i);<br>close();<br>}<br>selectWindow("Result of 0");<br>close();<br>setOption("BlackBackground", false); |
| Convert the HSB image in binary image (black and white) and fill holes in particles | run("Make Binary");<br>run("Fill Holes"); |
| Surround each particle | run("Create Selection"); |

**Step 5 : Analyze the particles of defined size and visualize particles on the image**

| | |
|---|---|
| Define the size of counted particles (tubercles from 0.005 to 20 mm²) to avoid false particles (too small or too big). This is done empirically as for color threshold. It opens the ROI manager that contains all selected particles. | run("Analyze Particles...", "size=0.05-20 summary add"); |
| The cropped image is opened | open(newdir+"\\"+name+"_crop.tif"); |
| And the selected particles (Regions Of Interest based on color threshold and size) are applied on the cropped image | roiManager("Show All"); |

**Step 6 : Count particles and obtain an image with surrounded particles**

| | |
|---|---|
| The number of particles (ROI) is counted to be reported in the table « tubercle number » | roi=roiManager("count"); |
| Activate the window of « tubercle number » table | selectWindow("tubercle number"); |
| Create column named « image name » to indicate the name of the image analyzed | Table.set("image name",j-1,name); |
| Create column « tubercle.number » and affiliate the number of tubercles counted in this image, from data in the ROI manager | Table.set("tubercle.nbre",j-1,roi); |
| Update the table | Table.update; |
| The following operations are conditioned by the fact that the ROI value is superior to 0 | if(roi>0) |
| Open a new loop | { |
| Save ROI manager data (all the counted particules) | roiManager("Save", newdir+"\\"+name+".zip");<br>roiManager("Measure"); |
| Surround particles on the cropped image | roiManager("Show All");<br>run("Flatten"); |
| Save this new image in the Results folder with its original name and « _flatten » suffix | saveAs("tiff",newdir+"\\"+name+"_flatten"); |
| Save the measurements (area and center of mass) of surrounded tubercles in an excel file | saveAs("Results",newdir+"\\"+name+"_Results.xls"); |
| End of the conditionned operations, end of the loop. | } |
| Close all opened windows | run("Close All"); |

| | |
|---|---|
| Re-initiate the ROI manager<br>End of the loop and go for next image | roiManager("Reset");<br>} |
| **Step 7 : A final excel table summarizes the number of tubercles for each image** | |
| Once all the images of the folder have been analyzed (tubercles counted), the loop stops and « analysis is done » appears on the screen<br>Select the window « tubercle number ») | <br><br><br>selectWindow("tubercle number"); |
| Save it as an excel format, summing up all the results obtained for each image | Table.save(newdir+"\\"+"tubercle number.xls"); |
| Then close it | selectWindow("tubercle number");<br>run("Close"); |
| Select window « Results »<br>And close it | selectWindow("Results");<br>run("Close"); |
| Select « ROI manager » window<br>And close it | selectWindow("ROI Manager");<br>run("Close"); |
| All the opened windows are closed<br>Message to inform user that analyze is done | run("Close All");<br>waitForUser("The end"," analysis is done"); |

**3b :** Semi-Automatic counting with manual options.

Some steps can be done manually for each image, such as defining the scale, cropping the image, adding or suppressing tubercles. In that case, manual command can be inserted in the above macro in place of an automatic command.

| | |
|---|---|
| **Manual scale (replace Step 2)** | |
| Select line tool on the ImageJ interface | setTool(« line ») ; |
| Open a dialog window named « Scale » in which is written « make a 50 mm line » | waitForUser(« Scale », « make a 50 mm line ») ; |
| Calibrate the image | run(« setScale… », « known=50 unit=mm ») ; |
| **Manual cropping (replace Step 3)** | |
| Select the rectangle tool for cropping on the ImageJ interface | setTool(« rectangle ») ; |
| Open a dialog window named « Crop » in which is written « define the region of interest» which allows the user to define manually the region of the image to be analyzed by tracing a rectangle containing all the | waitForUser(« Crop », « define the region of interest ») ; |

| | |
|---|---|
| tubercles<br><br>Crop the image and save the cropped image in the « Results » folder with the name of the original image and « _crop » suffix | <br>run(« Crop ») ;<br>saveAs(« tiff »,newdir+ « \\ » +nom+ « _crop ») ; |
| **Adjusting tubercle number visually by addition and suppression (before Step 6)** | |
| Activate the oval tool on the ImageJ interface to surround additional tubercle<br><br>Open a dialog window « additional tubercles » with instruction to suppress or add tubercle directly on the image and increase analysis accuracy.<br><br>To continue the macro, press the enter key | setTool (« oval ») ;<br><br><br>waitForUser("Adjust number of tubercles", "To suppress false tubercle: select the tubercle on the image and press the suppress key. To add a Tubercle: surround it on the image and press the "t" key.<br>Click "OK" when finish;"); |
