# Supplementary material for: Image analysis for the automatic phenotyping of Orobanche cumana tubercles on sunflower roots
Source: Plant Methods. 2021 Jul 21;17:80. doi: 10.1186/s13007-021-00779-6 (PMC8293553; doi:10.1186/s13007-021-00779-6)
Supplement: Supplementary file 6 — Additional file 6. Differences between manual counting and automatic macrocounting of the number of tubercles. [file 13007_2021_779_MOESM6_ESM.pdf]

Additional File 6

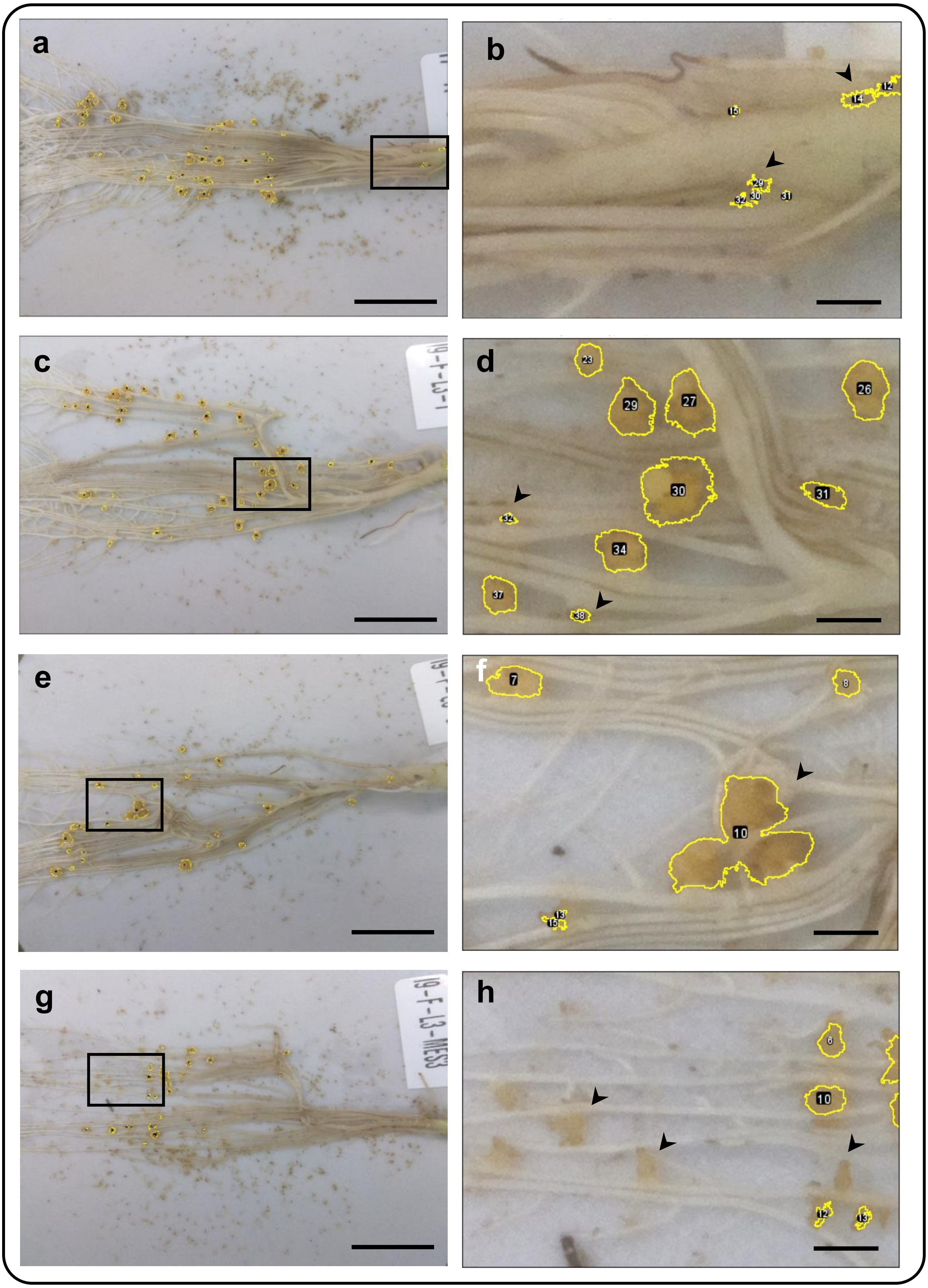

Differences between manual counting and automatic macrocounting of the number of tubercles.

Three week-old root images of the sunflower genotype 2603 inoculated with the race Bourret on the left, and image magnification on the right. Automatic macro counting of tubercles counted additional tubercles compared to manual counting because of counting false tubercles in the stem base (**a-b**), detection of very small tubercles (not detected by eye, (**c-d**). Automatic counting assembled group of close tubercle as only one particle (**e-f**) and did not count pale tubercles (**g-h**). Bar = 2 cm (**a**, **c**, **e**, **g**). Bar = 2,5 mm (**b**,**d**, **f**, **h**).
