# Supplementary material for: Image analysis for the automatic phenotyping of Orobanche cumana tubercles on sunflower roots
Source: Plant Methods. 2021 Jul 21;17:80. doi: 10.1186/s13007-021-00779-6 (PMC8293553; doi:10.1186/s13007-021-00779-6)
Supplement: Supplementary file 7 — Additional file 7. Phenotyping of 5 sunflower genotypes with 2 O. cumana races 21 days post inoculation using RhizOSun. [file 13007_2021_779_MOESM7_ESM.pdf]

Additional File 7

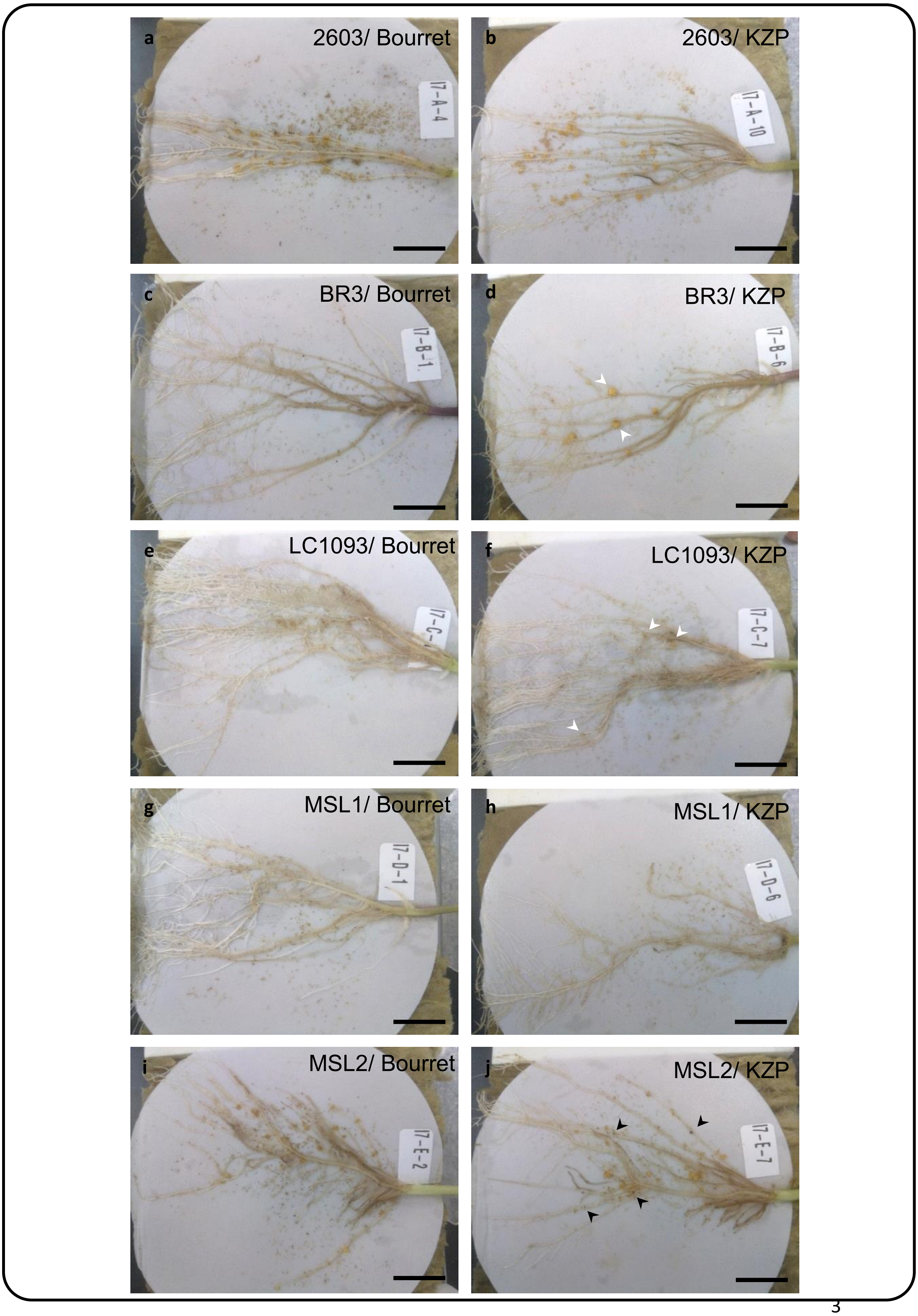

Phenotyping of 5 sunflower genotypes with 2 *O. cumana* races 21 days post inoculation using RhizOSun.

On the left side, inoculation with the *O. cumana* race Bourret. On the right side, inoculation with the *O. cumana* race KZP. **a-b**: the susceptible genotype 2603 to both *O. cumana* races. **c-d**: the genotype BR3 resistant to Bourret (no tubercles) and susceptible to KZP (well-developed tubercles, white arrowheads). **e-f**: the genotype LC1093 resistant to Bourret and tolerant to KZP (low number of tubercles, white arrowheads). **g-h**: the genotype MSL1 resistant to both *O. cumana* races. **i-j**: the genotype MSL2 susceptible to Bourret and late resistance to KZP with appearance of necrotic tubercles (black arrowheads). Bar = 20 mm.
