# Supplementary material for: Image analysis for the automatic phenotyping of Orobanche cumana tubercles on sunflower roots
Source: Plant Methods. 2021 Jul 21;17:80. doi: 10.1186/s13007-021-00779-6 (PMC8293553; doi:10.1186/s13007-021-00779-6)
Supplement: Supplementary file 8 — Additional file 8. Distribution of the number of tubercles/ rhizotron for 5 sunflower genotypes inoculated with 2 races of O. cumana, data obtained by manual counting. [file 13007_2021_779_MOESM8_ESM.pdf]

Additional File 8

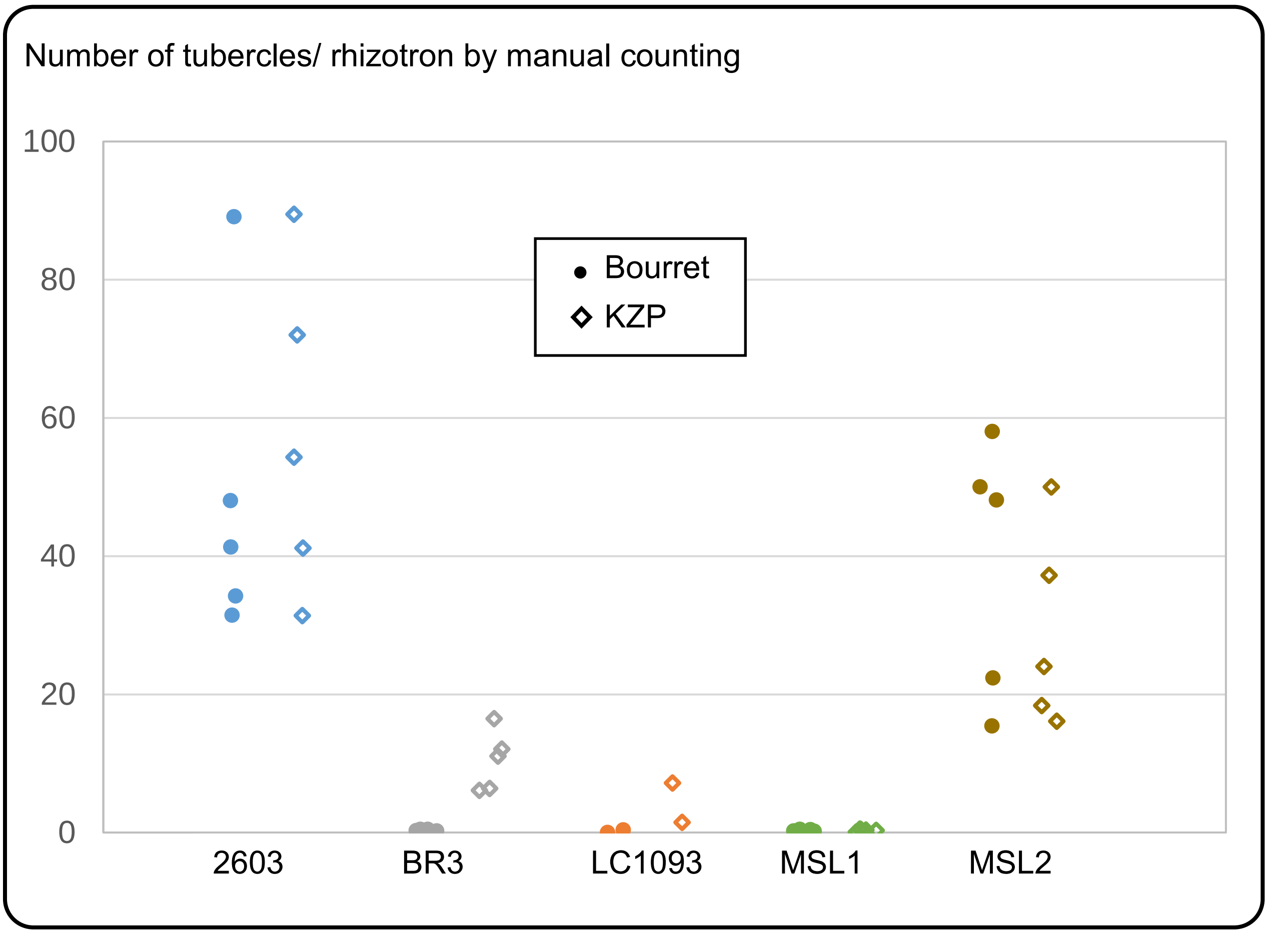

Distribution of the number of tubercles/ rhizotron for 5 sunflower genotypes inoculated with 2 races of *O. cumana*, data obtained by manual counting.

For each sunflower genotype, the numbers of tubercles/ rhizotron counted by manual counting are represented by full circles for the race Bourret (on the left side) and empty rhombus for the race KZP (on the right side).
